# Trends and Potential of Machine Learning and Deep Learning in Drug Study at Single-Cell Level

**DOI:** 10.34133/research.0050

**Published:** 2023-03-09

**Authors:** Ren Qi, Quan Zou

**Affiliations:** ^1^ Yangtze Delta Region Institute (Quzhou), University of Electronic Science and Technology of China, Quzhou 324000, China.; ^2^ School of Life Science and Technology, University of Electronic Science and Technology of China, Chengdu, China.; ^3^ Institute of Fundamental and Frontier Sciences, University of Electronic Science and Technology of China, Chengdu, China.

## Abstract

Cancer treatments always face challenging problems, particularly drug resistance due to tumor cell heterogeneity. The existing datasets include the relationship between gene expression and drug sensitivities; however, the majority are based on tissue-level studies. Study drugs at the single-cell level are perspective to overcome minimal residual disease caused by subclonal resistant cancer cells retained after initial curative therapy. Fortunately, machine learning techniques can help us understand how different types of cells respond to different cancer drugs from the perspective of single-cell gene expression. Good modeling using single-cell data and drug response information will not only improve machine learning for cell–drug outcome prediction but also facilitate the discovery of drugs for specific cancer subgroups and specific cancer treatments. In this paper, we review machine learning and deep learning approaches in drug research. By analyzing the application of these methods on cancer cell lines and single-cell data and comparing the technical gap between single-cell sequencing data analysis and single-cell drug sensitivity analysis, we hope to explore the trends and potential of drug research at the single-cell data level and provide more inspiration for drug research at the single-cell level. We anticipate that this review will stimulate the innovative use of machine learning methods to address new challenges in precision medicine more broadly.

## Introduction

Drug study is a very important problem in the cancer community, and many tasks aim to ultimately increase the effectiveness of cancer drugs by looking at the impact of the drug and its resistance on cancer cells [1]. The focus of drug development studies includes understanding drug response, discovering new drugs, predicting interactions between drugs and target diseases, and ranking drugs according to their potential [2]. These studies are important to develop new and effective treatments for various diseases. All these studies aim to improve the quality of life by reducing the burden of diseases and making treatments more effective.

Concentrating on the above aspects could provide valuable insights into the understanding of drug biomechanism [3]. However, considering the complexity of the disease, individual differences, and the huge number of drug compounds, it is almost impossible to discover drugs experimentally [4]. Thus, it is necessary to apply machine learning (ML) and deep learning (DL) technologies to drug response studies so as to save development costs and time [5,6].

At present, there are enough public data on disease cell lines to develop genomic signatures. For example, Genomics of Drug Sensitivity in Cancer (GDSC) [7], Cancer Cell Line Encyclopedia (CCLE) [8], the Cancer Therapeutic Response Portal (CTRPv2) [9], and the National Institutes of Health Library of Integrated Network-based Cellular Signatures Connectivity Map (NIH LINCS CMap L1000) [10] are the most popular drug screens in cancer cell line databases. These cancer cell line data can be used to develop better treatments for diseases and to improve our understanding of how diseases develop. Besides, more research is needed to mine data information.

ML and DL technologies make it possible to screen cell lines against thousands of chemical compounds, both clinically approved and experimental [11]. As a result, these public cancer cell line datasets spurred the development of predictive models. ML technologies can help identify new therapeutic targets and optimize existing treatments. Currently, numerous ML methods, especially DL models, have been developed to make drug response predictions [12]. For example, SCATTome is an ML model that combined random forest (RF) [13], LASSO [14], and support vector machine (SVM) [15] to predict drug sensitivity on targeted gene expression profiling of single cells within human myeloma tumors [8].

However, due to tumor cell heterogeneity, drug resistance and low efficiency are always challenging problems in clinical treatments [16]. If we can successfully predict the cell–drug response at the single-cell level, it will greatly influence the way cancer patients use drugs, which in turn will somehow provide great benefits for future cancer patients [17]. Not only would the outcome greatly benefit the health of cancer patients but also cancer occurs at the cellular level [18]. Studies look at cancer cell drug resistances, cancer drug side effects, and cancer drug sensitivities to measure the right outcomes, so it has the high potential to make way for cancer advancements [19].

Fortunately, we have noticed some progress in drug response studies at the single-cell level. For example, scGen used variational autoencoder (VAE) and latent space vector arithmetic to capture features and successfully distinguish responding from nonresponding genes and cells on single-cell gene expression data [20]. Besides, DeepHACKS integrated bidirectional long short-term memory (LSTM), conventional ML, and an autoencoder model to implement temporal feature extraction and drug response prediction [21]. These methods demonstrate that DL can help predict drug response at the single-cell level. ML technologies are revolutionizing drug discovery and development, and hold great promise for the future of personalized medicine. Ultimately, ML and DL technologies have the potential to improve the lives of patients by providing more effective and tailored treatments.

However, to date, drug response studies at the single-cell level lag behind relatively, and most of the published review literature focuses on the comparison of developed ML on cancer cell line data [129–131]. This is due, in part, to the challenge of measuring drug response at the single-cell level. The challenge of studying drug response based on single-cell sequencing data is that there are not enough single-cell sequencing data and cells’ responses after drug exposure [22]. Obviously, delays during the lack of collection of necessary data information would affect the conduct of research.

In this review, we focus more on DL techniques with a much more detailed description and method comparison in the field of drug study. First, to better understand the process of drug development, we provide a workflow about drug discovery and development and show the role of ML methods in the overall pipeline. Further, we introduce ML methods following the analysis pipeline and public data format and cell line databases in cancer. As a comparison, we also present the limited publicly available single-cell datasets of cancer. Besides the data and models, we also summarize the applications on both cancer cell lines and single-cell data of ML and DL methods in drug response, drug discovery, interaction prediction among drug–target–disease, and so on. Notably, to compare the technical application gap between single-cell sequencing data analysis and cancer single-cell drug sensitivity analysis, we systematically summarized the single-cell sequencing data analysis methods. Finally, we conclude the advantages and disadvantages in the field of drug studies, then discuss the challenges and opportunities of predicting drug sensitivity at the single-cell level, and give the potential direction for future studies on cancer single cells.

## General Methods and Workflows for ML and DL in Drug Study

### An overview of ML and DL methods and their model training

**Fig. 1.**
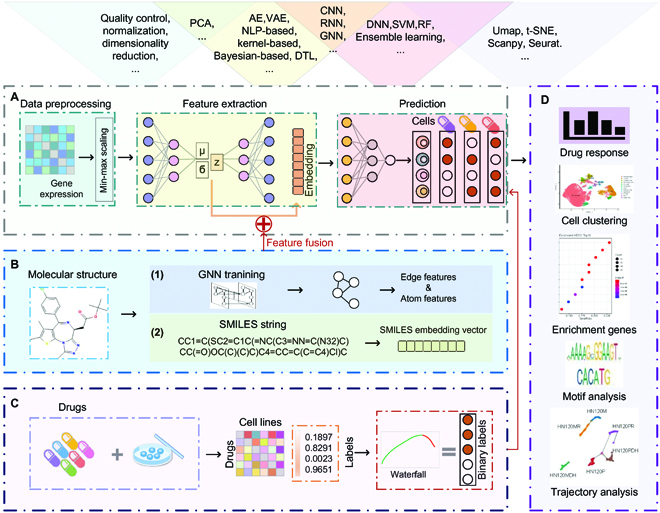
(A to D) ML training procedures and downstream analysis workflow. SVM, support vector machine; RF, random forest; NLP, natural language processing.

**Fig. 2.**
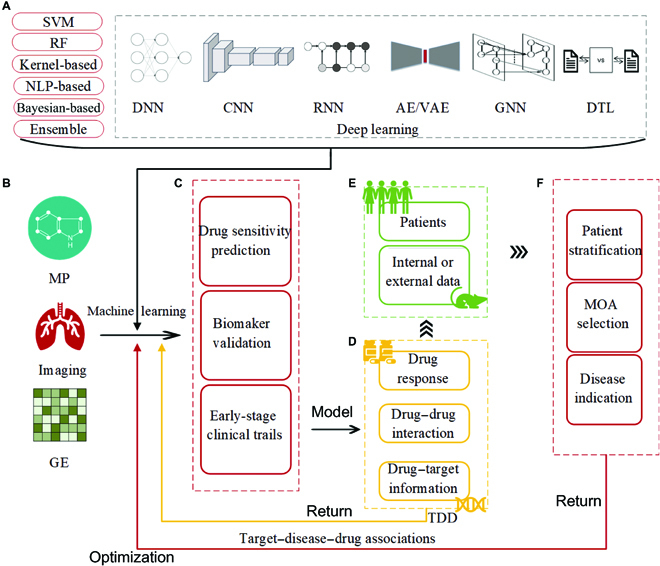
(A to F) Drug response and development workflow using ML technologies. SVM, support vector machine; RF, random forest; NLP, natural language processing; MP, molecular profiling; GE, gene expression data; MOA, mechanism of action; TDD, target–disease–drug associations.

Common conventional ML contains kernel-based [23], Bayesian-based [24], and NLP-based methods [25], SVM [15], RF [13], and Ensemble learning [26,27]. Here, Ensemble learning refers to a class of strategies where, instead of building a single model, multiple base models are combined to perform tasks [27]. DL methods in Figs. 1 and 2 contain deep neural network (DNN), convolutional neural network (CNN), recurrent neural network (RNN), auto-encoder or variational auto-encoder (AE/VAE), graph neural network (GNN), deep transfer learning (DTL), and other variants. Specifically, autoencoders have been used to impute missing data [28], extract gene expression signatures [29], and detect expression outliers in microarray data and RNA sequencing (RNA-seq) gene expression data [30]. Especially in biological data, there are a lot of unlabeled data, but they are related to very important issues that need to be studied.

A DNN is a multilayer unsupervised neural network with multiple nonlinearly mapped feature transformations that can be fitted to highly complex functions. DNNs use the output features of the previous layer as the input of the next layer for feature learning, and learn a better feature representation of the existing input by mapping the features of the existing space samples to another feature space after layer-by-layer feature mapping. Here, we briefly introduce the main structure and training process of a basic neural network. A most basic neural network structure contains an input layer, a hidden layer, and an output layer [31]. Generally, data are first input in the input layer of a DNN, then the data are mapped through several nonlinear hidden layers, and the result is finally obtained in the output layer by a suitable activation function. The training process of the network consists of 3 parts: the first part is data partitioning. The general dataset is divided into 3 subsets, namely, the training set for optimizing the model parameters, the validation set for verifying the model performance, and the test set for the final evaluation of the model performance. The second part is parameter fitting. The initial values of the model parameters of the neural network can be random, and then the parameters of the model are optimized iteratively using the gradient descent method. The third part is the model evaluation. Here, the model has been trained and the hyperparameters have been trained fixed by the data in the validation set, and this step is to evaluate the performance of the trained model on the test set.

To summarize, there are 3 core ideas of DNNs: (a) unsupervised learning is used to pretrain each layer of the network; (b) unsupervised learning uses the output of the previous layer as the input of the next layer to train each layer by layer; and (c) finally, all layer parameters are fine-tuned by a classifier (supervised learning). The above training process is the basic operation of neural networks, and we can select the appropriate neural network model according to the task objective and continuously reduce the loss between predicted and true labels through model training. Specifically, among all neural network models, DNN and AE/VAE are the most basic and widely used, and they can be integrated and inserted into other models. In addition, CNN is often applied to image data analysis, RNN can be useful in context-dependent sequence data analysis, GNN mines data association through graph theory, and DTL performs feature migration through adversarial learning among neural networks [32]. Therefore, we can select the appropriate neural network for cell classification, disease detection, and sequence analysis according to the task type in practical applications [28].

### ML and DL method training procedures and downstream analysis workflow

In general, ML methods can model gene expression features and cell–drug responses (Fig. 1). Here, we list the commonly used method models in different step categories above Fig. 1A. First, gene expression data are generally preprocessed before being input to ML training models (Fig. 1A), and since high-throughput single-cell sequencing data contain a large number of low-quality genes, we need to perform quality control on single-cell sequencing data to remove low-quality genes. In addition, the gene dimension of single-cell sequencing data is large relative to the number of cells, and dimensionality reduction is usually performed before feature extraction. Second, ML models need to perform feature extraction. There are many methods for feature extraction, such as AE, VAE, kernel-based methods, and GNN. The low-dimensional features obtained by feature extraction are high-level features, which can characterize the gene expression of cells. Besides gene features, the molecular structure of drug compounds can also be helpful as features for the prediction of cell–drug responses. Here, GNN can be used to learn the edge and atomic features in the graph structure of drug compounds on the one hand, and to transform drug compound molecules into vector features on the other hand (Fig. 1B). Multiple types of features can work together through feature fusion. In the model prediction step, we can obtain label information of cell–drug responses from publicly available datasets (Fig. 1C), and then ML models can use the gap between the true label and the predicted label to train a correction on the extracted features and derive the gene expression features that are most associated with drug responses. Finally, the trained ML model not only is able to study the relationship between gene expression and drug response but also can perform cell clustering analysis, enrichment gene analysis, motif analysis, and pseudo-time trajectory analysis by the extracted feature genes (Fig. 1D).

**Fig. 3.**
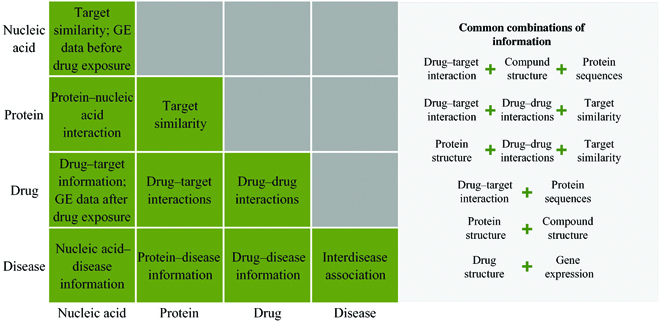
Existing drug–target–disease information, interaction, and combinations. Data information as input could be nucleic acid sequences, protein, drugs, disease, and interactions among them. Names of data information and interaction are listed accordingly.

Besides, Fig. 2 illustrates the role of a trained ML model in the overall drug research process. Usually, different ML approaches require different types of data features as input. In drug research, the data used as input can be nucleic acids, proteins, drugs, diseases, and information about their interactions. In general, indispensable input data are gene expression matrices of cells and labels of cell–drug responses. Figure 3 shows common data information and combinations as input in drug study. Here, Fig. 2B gives information about the data that may be input in a drug study. Assuming that the data related to drug studies are trained using the ML approach in Fig. 2A, the resulting models can be used to predict drug sensitivities, validate biomarkers, etc., and the resulting models and conclusions from training for different research purposes can also be used to conduct early clinical trials (Fig. 2C). In addition, trained ML models can be used to facilitate the discovery of new functions of drugs (Fig. 2D). For example, the RNN in a neural network model can feed back information on drug–drug interactions (DDIs) or drug–target binding inferred by the ML model to the ML training, and this process can facilitate further refinement of the ML model. Further, we can take the drug information mined by training refinement (Fig. 2D) and experimentally validate it with patient or internal or external data (Fig. 2E). This process can facilitate the implementation of different treatment options for different patients and help clinical staff to determine different mechanisms of action and disease indications (Fig. 2F). Finally, feedback from the clinical data can also be used to optimize ML training. It is only through iterative training and continuous access to feedback from the model and the clinic that the model can continue to drive the overall development of drug research.

Furthermore, new disease mechanisms can be identified using ML methods for drug studies, which will help discover key genes mutated in tumor cells, while trajectory analysis of gene expression patterns can find upstream regulators of drug resistance genes, which in turn can identify new therapeutic targets. Clinics can predict drug response based on the correlation of different single cells, using common key genes as potential targets for drug intervention. ML approaches may enable a shift in cancer drug design and use, and help to address key issues such as the continued decline in drug action in cancer drug development. Besides, approaches have the potential to provide new insights into the mechanisms of drug action and resistance. The model generated by ML approaches can be used to develop new drugs and to optimize existing treatments.

### Genomics data with drug response information

#### Cancer cell line data with drug response information

**Fig. 4.**
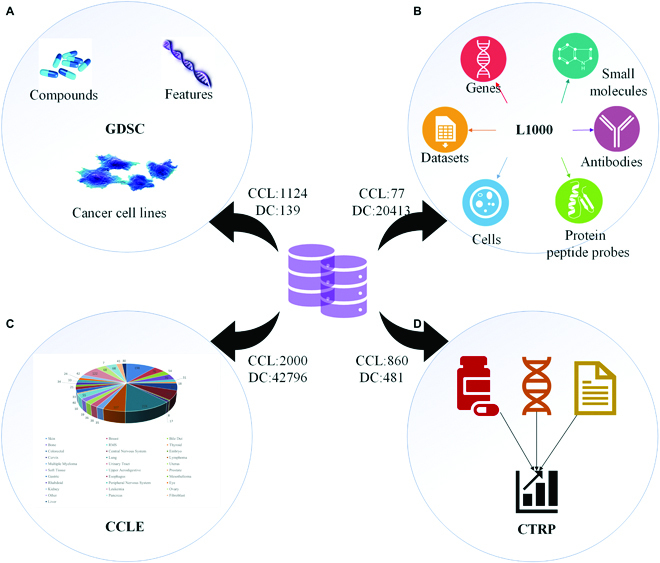
(A to D) Platforms of cancer cell lines screened with compounds. These platforms provide drug response data, genomic markers of sensitivity, and basic processing functions for biomarker discovery. CCL, cancer cell lines; DC, drug compounds.

GDSC [7] contains 860 human cancer cell lines and 481 drug compound combinations. Information can be searched by drug, gene, or cell line name. Users can also browse all cancer features, cell lines, and compounds at https://www.cancerrxgene.org/. For example, GDSC provides targets, pathways, relative datasets, and cell line half-maximal inhibitory concentration (IC_50_) and Area Under Curve (AUC) values in the part of compounds. We found that GDSC was the most used cell line database in research (Fig. 4A).

L1000 [10] profiled 1,000 landmark genes in 77 human cell lines with drug treatment (Fig. 4B). The latest version can search for information by datasets, small molecules, cells, genes, proteins, and antibodies. Besides, L1000 provides a KINOMEscan kinase small-molecule binding assay to test if molecules bind the kinase. All information is obtained from the portal https://lincsportal.ccs.miami.edu/dcic-portal/.

CCLE [8] project provides the genetic and pharmacologic characterization of many human cancer models. In the diseased part, the CCLE portal presents cell line, primary disease, tumor type in cell lines in disease, and gene, compound, dataset, *t* statistic, and *P* value in dependencies enriched in disease. In the compound part, it provides relative sensitive cell lines, enriched lineages, top correlated expression, datasets with data for a certain compound, and metadata (https://depmap.org/portal/ccle/). We showed diseases included in CCLE and their corresponding cell line numbers in Fig. 4C.

CTRPv2 [9] contains 860 cell lines and 481 drug compound combinations (Fig. 4D). Functions of this portal contain annotations for small molecules and cancer cell lines (CCls), interactive interface, enrichment, correlation analysis, visualization, scatterplots and curves, and filters (https://portals.broadinstitute.org/ctrp.v2.1/).

While bulk RNA-seq expression data reflect the average expression of genes in all cells of a piece of tissue, single-cell sequencing reflects the gene expression of each cell in the tissue, so single-cell sequencing data have a precision that cannot be replaced by bulk sequencing data.

#### Single-cell data with drug response information

Currently, single-cell sequencing technologies can be divided into 2 categories according to the counting method. The first category is full-length sequencing represented by smart-seq. This double-end sequencing is suitable for studies such as embryonic development because it sequences complete transcripts with high accuracy, although the cell count throughput of the sequenced cells is low. The other type is the more common 3' end or 5' end sequencing, which usually has higher throughput because it sequences only the ends of transcripts and is the more commonly used single-cell sequencing technique today.

With the development of single-cell sequencing technology, the volume of single-cell sequencing data has grown exponentially. The database that stores the most single-cell data is the Gene Expression Omnibus (GEO) database. The GEO database collects a rich amount of single-cell data, and the data types are relatively standardized and have more detailed original experimental designs and experimental procedures described. Although many single-cell data resources already exist, most of the single-cell sequencing data do not provide drug response labels. In this article, we intend to contribute to the research of precision medicine from the perspective of precision of single-cell sequencing data, so we found 27 cancer single-cell sequencing data with drug response labeling information from publicly released cancer single-cell sequencing data of different species and data sources.

Table 1 shows the collected publicly available cancer single-cell datasets with drug responses. These data include 13 diseases such as acute myeloid leukemia, breast cancer, chronic lymphocytic leukemia, melanoma, and circulating tumor cells in human or mouse organisms. However, the information collected for most single-cell datasets remains incomplete. Drug response prediction studies at the single-cell level face great challenges due to the presence of these problems such as gene expression before or after exposure to drug compounds only or the lack of some information on the duration of drug exposure. Details of the cancer single-cell datasets with drug response labels are shown in Table 1.

**Table 1.** Publicly available cancer single-cell data with drug response information.

Index	Sample	Organism	Cancer	Drug	DOI
GSE122843 [74]	6	M	AML	IBET	10.1038/s41467-019-10652-9
SRP114962 [75]	6,633	H	Breast cancer	Paclitaxel	10.1016/j.cell.2018.03.041
GSE122336 [76]	21	M	Breast cancer	Trastuzumab	10.1038/s41467-019-11729-1
GSE111014 [77]	188	H	CLL	Ibrutinib	10.1038/s41467-019-14081-6
GSE111015 [77]	12	H	CLL	Ibrutinib	10.1038/s41467-019-14081-6
GSE116237 [78]	865	H	Melanoma	Dabrafenib, trametinib	10.1016/j.cell.2018.06.025
GSE117872 [79]	1,431	H	OCC	Cisplatin	10.1038/s41467-018-07261-3
GSE107864 [80]	189	H	Breast cancer	E2 treatment	10.1016/j.celrep.2018.10.093
GSE119455 [80]	39	H	Breast cancer	E2 treatment	10.1016/j.celrep.2018.10.093
GSE104987 [81]	3	H	Breast cancer	Endocrine	10.1016/j.ccell.2018.10.014
GSE114462 [1]	11	H	Breast cancer	Bortezomib	10.1038/s41586-018-0409-3
GSE117309 [82]	10	H, M	Breast cancer	Tamoxifen	10.1038/s41588-019-0424-9
GSE122233 [83]	757	M	CTC	BET, JQ1	10.1073/pnas.1814102116
SRP106621 [84]	114	H	ESC	Paclitaxel	10.1016/j.canlet.2018.01.059
GSE121107 [85]	3	H	FPRMS	Vorinostat, LSD690	10.1038/s41467-019-11046-7
GSE127298 [86]	224	H	Leukemia	MMI	10.1016/j.ccell.2019.11.001
GSE112274 [87]	1,090	H	Lung cancer	TKI	10.1373/clinchem.2018.295717
GSE69405 [88]	2	H	Lung cancer	Multiple	10.1186/s13059-015-0692-3
GSE108397 [89]	337	H	Melanoma	BRAF inhibitor	10.1101/gr.234062.117
GSE108394 [89]	2	H	Melanoma	BRAF inhibitor	10.1101/gr.234062.117
GSE115978 [90]	7,186	H	Melanoma	CDK inhibition	10.1016/j.cell.2018.09.006
GSE97681 [91]	122	H	Melanoma	Vemurafenib	10.1038/nature22794
GSE72056 [92]	4,645	H	Melanoma	Vemurafenib, Dabrafenib.	10.1126/science.aad0501
GSE110894 [74]	1	M	LSC	IBET	10.1038/s41467-019-10652-9
GSE129730 [93]	15	M	Medulloblastoma	Vismodegib	10.1038/s41467-019-13657-6
GSE140440 [94]	324	H	Prostate cancer	Docetaxel	10.1158/1541-7786
GSE149383 [95]	33	H	Melanoma	Crizotinib, EGFR-TKI	10.1038/s41467-021-21884-z

H, *Homo sapiens*; M, *Mus musculus*; AML, acute myeloid leukemia; CLL, chronic lymphocytic leukemia; CTC, circulating tumor cells; ESC, esophageal squamous cancer; OSC, oral squamous cell carcinoma; TKI, tyrosine kinase inhibitor; LSC, leukemia stem cells; MMI, menin-MLL inhibitor.

## ML and DL Techniques Can Accelerate Drug Study

**Fig. 5.**
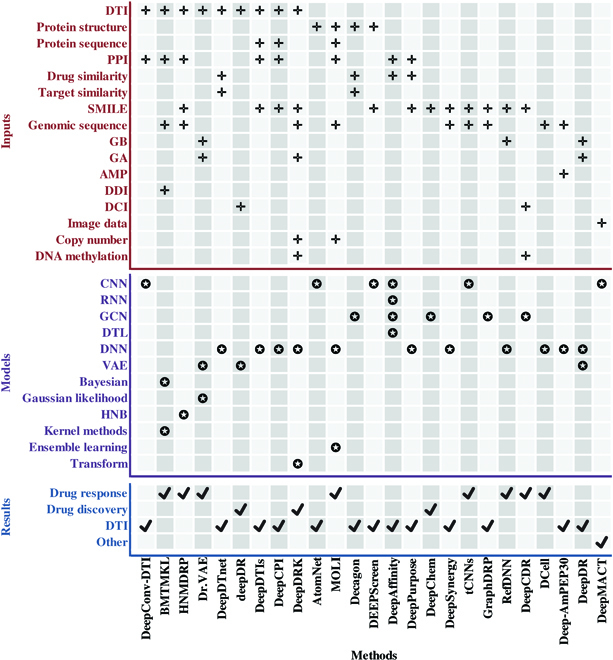
ML tools and their required data characteristics and functions in the drug study. DTI, drug–target information; PPI, protein–protein interaction; SMILE, simplified molecular-input line-entry system; GB, gene expression data before drug exposure; GA, gene expression data after drug exposure; AMP, antimicrobial peptide sequence; DDI, drug-disease information; DCI, drug chemical information; HNB, heterogeneous network-based.

The study of cancer single cell sequencing data is critical to understanding the complexity of the cancer genome and facilitating the development of precision medicine. The clinical outcome, drug resistance, and cancer metastasis recurrence are all related to the heterogeneity of cancer cells. However, there are few cancer cell gene expression and drug response cancer studies studied at the single-cell level, most of the existing studies have been performed at the bulk level, and many computational methods have been developed for analyzing cancer cell lines with drug responses. Therefore, this paper provides a review of ML and DL techniques and related tools for drug response analysis, at both the cancer cell line and single-cell level. These literature include different types of tasks, including drug discovery, drug response, and drug interaction studies. Among the literature we collected, 9 of the models require drug target information as input, 4 tools require protein structures, 6 methods require drug molecules as input, and 2 additional methods require protein sequences. Furthermore, by summarizing the literature in Fig. 5, we found that the simplest DNN is the most frequently used model in drug research. Figure 5 provides a detailed summary of the models used by ML methods during drug research, the data features that require input, and the functions implemented by the models.

### Drug discovery

There are 2 main approaches to drug discovery: phenotypic screening and molecular target-based approaches [33]. Phenotypic screening is mainly used to identify drugs that have a desired effect on a particular disease or condition. Molecular target-based approaches are used to identify drugs that bind to a specific target protein or enzyme. Phenotypic screening is a powerful tool for identifying new drug candidates. For example, penicillin was serendipitously found by phenotypic activity [34]. Many models were developed on large amounts of RNA-seq data with drug response to evaluate the drug sensitivity of various cancer subpopulations given their gene expression profile [35]. These approaches help to identify new therapeutic targets and to evaluate the potential of new drugs. For example, RACS is a ranking system for predicting the potential anti-cancer synergy of drug combinations [36]. The method used manifold ranking and integrated multiple features extracted from a breast cancer cell line. DeepMACT presented a new DNN model for the quantification of cancer metastases and antibody targeting by embedding CNNs and autoencoder [37]. In DL methods, all DNNs apply different parameters across different input features, and CNNs apply the same filter at each position.

Pharmacogenomic data are multisource and multiomics, including genomics, drug–target interaction (DTI), and epigenomics. DeepDRK provided a kernel-based method that could integrate these data and transfer information [38]. KRL also used kernel learning, but it was for personalized drug recommendations [39]. Heterogeneous biological networks have been applied in drug repurposing. Here, deepDR learned features of drugs by a multimodal deep autoencoder that integrated 10 networks, and then according to drug-disease information infer drug candidates by VAE [40].

Among all DL networks, the graph convolutional network (GCN) is prevalent in drug discovery. GCNs apply the same set of parameters to each node and, in combination with data of fingerprint descriptors, have made features extracted from graph structure more meaningful and explainable. Yu et al. [41] successfully ensembled GCN and ML models to classify central nervous system (CNS) drugs with pretty much prediction accuracy. Besides, the GCN model in the DeepChem framework was for drug discovery [42]. These approaches are complementary and together can provide a more comprehensive coverage of the drug discovery space.

### Drug response

The concept of using DL to discover observations on drug sensitivity in single-cell sensitivity is innovative [22]. DL has the potential to help researchers better understand how cells respond to drugs and identify new targets for therapeutic intervention. It is necessary to train and test models of drug response prediction before real-world validation and potential for clinical translation. Predicting drug sensitivity at the single-cell level will bridge the gap between basic research on drug discovery and clinical treatments [43]. The success of relative studies will help clinicians to exclude low-sensitive drugs in practice or even choose more effective drug treatments for different cancer subpopulations with more information provided. Of note, there are 2 DL methods developed for drug response single-cell sequencing data [20,21].

Costello et al. [44] proposed an ML method named BMTMKL for predicting therapeutic responses from genomic, proteomic, and epigenomic profiling data. The method used Bayesian multitask multiple kernel learning algorithms and tested drug compound responses in human breast cancer cell lines. It is remarkable that BMTMKL performs best in the NCI-DREAM (National Cancer Institute-Dialogue for Reverse Engineering Assessments and Methods) Drug Sensitivity and Drug Synergy Challenge for drug response prediction competition with a weighted average of the probabilistic concordance index (wpc-index) of 0.583 [131]. Heterogeneous Network-based Method for Drug-Response Prediction (HNMDRP) predicted drug responses based on heterogeneous networks, which contain cell line similarity network, drug similarity network, target similarity network, and DTI network [45]. Data information input to the model includes genomic sequences, drug compound structures, and target–disease–drug associations. It successfully predicted drug responses on 19 tissue types of GDSC dataset.

One trend in the drug study is the transition to advanced DL techniques for drug response predictions. To improve the drug response prediction accuracy, Sharifi-Noghabi et al. [46] present a DNN prediction model MOLI integrated multiomics, combining bulk mutation, copy number aberration, and genomic data. In addition, DCell distinguished the molecular mechanisms underlying genotype–phenotype associations by the DNN [47], where immunoglobulins are critical in disease regulation, which was important for drug resistance study. In addition, Choi et al. [48] proposed an innovative drug-based neural network model, named RefDNN. It researched anticancer drug resistance on GDSC and CCLE datasets. Notably, RefDNN could identify drug resistance biomarkers using trained weights of the network embedded in RefDNN. On the GDSC and CCLE datasets, RefDNN achieved 0.89 and 0.93 on the area under the curve of receiver operating characteristic curve (AUCROC) and area under the curve for the C peptide reactivity (AUCCPR) metrics, respectively, which were both better than HNMDRP. The experiments of RefDNN confirmed that the prediction accuracy of the proposed DNN model outperformed existing drug response prediction models that did not use DNNs.

In addition, tCNNs extracted drug features and genomic features from 2 CNN models, respectively, and then used phenotypic drug response prediction on GDSC cancer cell lines by a DNN model [49]. Inputted data are in simplified molecular input line entry specification (SMILES) and GDSC genomic format. GraphDRP does this also, but it established CDR prediction by GCN and DNN models, respectively [50], and results showed that GraphDRP outperforms tCNNS in all experiments. Results show that GraphDRP outperforms the method that uses only SMILE strings to represent drug properties in terms of root mean square error and Pearson correlation coefficient. This also indicates that it is more appropriate to consider the structure of the drug compound than to use the features of the string vector [50]. Moreover, DeepCDR used a hybrid GCN and multiomics data to study drug response [51]. It innovatively learned the latent representation of topological structures among drug compounds. Besides, deep generative networks have been pretty applied in the field of drug response prediction. Dr.VAE, Drug Response Variational Autoencoder, is such a generative and unified probabilistic ML model [52]. It predicted individualized drug response based on VAE and Gaussian likelihood models, and results on CTRPV2 and L1000 showed better prediction accuracy compared with benchmark ML methods.

Notably, there has been some progress in understanding drug response at the single-cell level, and DL models have also successfully predicted drug responses at subcellular and single-cell levels. Wang et al. [21] proposed an unsupervised ML framework DeepHACKS in 2022. The method integrated bidirectional LSTM, conventional ML, and an autoencoder model to implement temporal feature extraction and drug response prediction. Results showed that DeepHACKS can give a more precise and comprehensive quantification of the effects of drug actions and potential in various time-series data analysis applications. Recently, Chen et al. proposed scDEAL [128]. scDEAL is a framework for predicting cancer drug response at the single-cell level using DTL. The method predicts drug response by transfer models trained on bulk RNA-seq data to single-cell RNA-seq (scRNA-seq). This study inspired the discovery of drug treatment based on gene expression and cell perturbation response, and it has important implications for the treatment of cancer, as it suggests that a more personalized approach may be necessary. Additionally, this study highlights the potential of single-cell studies to improve our understanding of drug response.

There is great potential to use DL methods to study drug sensitivity at the single-cell gene expression level. Successful modeling of single-cell gene expression and drug response will lead to important clinical applications. Correlating drug response information at the single-cell level with transcriptome expression patterns in human cancers can help physicians design therapeutic regimens that are specific to the patient's condition. Predicting the most effective drug combinations at the single-cell sequencing level by ML methods can avoid treatment failure due to the heterogeneity of cancer cells.

### Drug interactions

#### Drug–target interactions

Identification of DTIs is important for drug discovery and development and helps us understand the mechanism of drug actions and off-target adverse events. CNN, DNN, NLP, and Boltzmann machine neural networks have been successfully applied in DTI predictions.

To identify interactions between known drugs and targets, DeepDTIs stacked multiple restricted Boltzmann machine neural networks [53]. DeepCPI is developed for drug discovery and repositioning. The model invoked natural language processing (NLP) techniques to extract features of drugs and then fit them into a multimodal DNN classifier to predict compound–protein interactions [54]. To reduce the barrier to applying DL technologies in drug–target interactions’ predictions, Huang et al. [55] designed a comprehensive and easy-to-use DL library, termed DeepPurpose. DeepPurpose combined with compounds, protein encoders, and over 50 neural network models, along with providing many other useful features to improve DTI prediction. The library modules contain protein and compound encoding, DTI prediction, and downstream prediction. Similarly, DeepConv-DTI also used CNN on raw protein sequences, but it trained the model by known DTIs and randomly generated DTIs as negative samples [56]. However, DEEPScreen produced high accuracy in DTI prediction by fitting a 2D drug structure into the CNN model to learn complex features [57].

#### Protein–protein interactions

CNN models demonstrate a capacity for inferring protein–protein interaction (PPI) by an image that exists in drug molecules or genomic sequences. For example, CNNC is a deep CNN for coexpression [58]. It innovatively represents each pair of genes as a histogram image and establishes inferring transcription factor gene and PPIs by CNNs. Karimi et al. [59] noticed the lack of methods that can predict drug–target affinity from sequences alone, and they proposed DeepAffinity unified graph CNN (GCNN) and RNN models. In another attempt to enhance PPI prediction, Deep-AmPEP30 applied CNN to predict short-length antimicrobial peptides from genomic sequences for drug discovery [60], and it achieves. Results in Deep-AmPEP30 manuscript indicate that grouping of amino acids is essential to detect correlations between amino acid properties and peptide functions. In the experimental part, Deep-AmPEP30 obtained the best results using PseKRAAC 86 features [Accuracy = 0.77, AUCROC = 0.82, The area under the precision-recall curve (AUPRC) = 0.8, kappa = 0.53, and Matthews correlation coefficient (MCC) = 0.54].

In addition to the CNN model, DNN, GNN, and embedding models have also been developed to make the PPI predictions. Specifically, DeepSynergy used a DNN model on drugs and genomics data, and it successfully predicted anticancer drug synergy [61]. DeepDTnet embedded drug–target, protein–protein, drug–drug, drug–disease, and drug–side effect heterogeneous networks to identify targets among known drugs [62]. DeepDTnet is highly accurate even without any drug target information, and the method has been approved by the U.S Food and Drug Administration to identify new targets for common drugs. It identified topotecan as an inhibitor that can be used directly on the human retinoic acid receptor [62]. Decagon constructed a multimodal graph of PPI, DTI, and the polypharmacy side effects for modeling and predicting polypharmacy side effects (AUROC = 0.872, AUPRC = 0.832) [63]. Table 2 lists the ML and DL methods with source code and in drug research applications, all of which were experimented on cancer cell lines.

**Table 2.** ML and DL methods with source code applied in the drug study at the bulk level.

Method	Platform	Functions	Source code
HNMDRP [45]	M,R	Drug response	https://github.com/USTC-HIlab/HNMDRP
Dr.VAE [52]	P	Drug response	https://github.com/rampasek/DrVAE
BMTMKL [44]	M,R	Drug response	https://github.com/mehmetgonen/bmtmkl
RefDNN [48]	P	Drug response	https://github.com/mathcom/RefDNN
DCell [47]	J,P	Drug response	https://github.com/idekerlab/DCell
tCNNs [49]	P	Drug response	https://github.com/Lowpassfilter/tCNNS-Project
MOLI [46]	P	Drug response	https://github.com/hosseinshn/MOLI
DeepCDR [51]	P	Drug response	https://github.com/kimmo1019/DeepCDR
GraphDRP [50]	P	Drug response	https://github.com/hauldhut/GraphDRP/
scDEAL [127]	P	Drug response	https://github.com/OSU-BMBL/scDEAL
DeepChem [42]	P	Drug discovery	https://github.com/deepchem/deepchem
DeepDRK [38]	R	Drug discovery	https://github.com/wangyc82/DeepDRK
deepDR [40]	P,M	Drug discovery	https://github.com/ChengF-Lab/deepDR
DeepDTIs [53]	P	DTI	https://github.com/Bjoux2/DeepDTIs_DBN
DeepCPI [54]	P	DTI	https://github.com/FangpingWan/DeepCPI
DeepDTnet [62]	P	DTI	https://github.com/ChengF-Lab/deepDTnet
DeepConv-DTI [56]	P	DTI	https://github.com/GIST-CSBL/DeepConv-DTI
DeepPurpose [55]	P	DTI	https://github.com/kexinhuang12345/DeepPurpose
DEEPScreen [57]	P	DTI	https://github.com/cansyl/DEEPScreen
DeepAffinity [59]	P	DTI	https://github.com/Shen-Lab/DeepAffinity
Deep-AmPEP30 [60]	P	DTI	https://cbbio.cis.um.edu.mo/AxPEP/
DeepSynergy [61]	P	DDI	https://github.com/KristinaPreuer/DeepSynergy
Decagon [63]	P	DDI	https://github.com/mims-harvard/decagon
KRL [39]	P	Other	https://github.com/BorgwardtLab/Kernelized-Rank-Learning
DeepMACT [37]	P	Other	http://discotechnologies.org/DeepMACT/
RACS [36]	R	Other	https://github.com/DrugCombination/RACS
Yu [41]	P	Other	https://github.com/tzuhuiyuatntu/cnsstudy2019

M, Matlab; P, Python; J, Java; DTI, drug–target interactions; DDI, drug–drug interactions.

#### Drug–drug interactions

Simple fully connected layers can obtain enough informative features and investigate the mechanism hidden behind drugs. For example, DDIMDL is a multimodal DL method that only applied fully connected layers and fused diverse cross-modality drug features, which contain chemical substructures, targets, enzymes, and pathways, to predict DDI-associated events [64].

Here are some works on drug interactions based on single-cell image data. Kandaswamy et al. [65] first applied both DNN and DTL to leverage single-cell image-based information in the analysis of breast cancer in 2016. They used DNN to map features of a single cell to a chemical mechanisms of action (MOAs) class and particularly saved the trained model to speed up parameter tuning steps by transfer learning when solving new MOA classification tasks. This model has boasted modest success in improving the classification accuracy of single cells and saving computational time. Then, Pavillon et al. [66] detected the state of macrophage activation at the single-cell level through an ML method. They build a multivariate statistical model based on penalized logistic regression on obtained morphological features on cell images by digital holographic microscopy (DHM) laser and molecular feature parameters by Raman spectra and assessed cell performance in a single-cell state. They discovered that morphological and Raman indicators are linked to the downstream phenotype and upstream intracellular molecular changes separately.

To evaluate single-cell response to drug exposure, Kobayashi et al. [67] present an ML model, combining maximum mean discrepancy (MMD) and kernel learning, and experiment on high-throughput bright-field imaging containing human breast adenocarcinoma cancer cells with and without drug exposure. They demonstrated that a single ML model had the power of inferring dose-dependent, drug-induced morphological change. Interestingly, Yanagisawa et al. [68] successfully classified the sensitivity of drugs by cell morphology during culture based on the CNN DL model VGG16 and predicted the response of anticancer drugs at the single-cell level. This study verified the possibility of finding effective antitumor drugs by using DL methods on circulating tumor cells extracted from blood. Novel insights obtained from cell tracking analyses have informed current drug treatment research and have led to the development of DL approaches to evaluate the impact of a drug on cell motility behaviors at the single-cell level. For example, Deep Tracking is a CNN model to extract features from the visual atlases, and it combines transfer learning with extracted features, which were encoded in the 2D visual images of cell trajectories, to classify the cell motility behaviors [69].

## Advanced Studies in General Single-Cell Data Analysis Can Inspire Drug Research

**Fig. 6.**
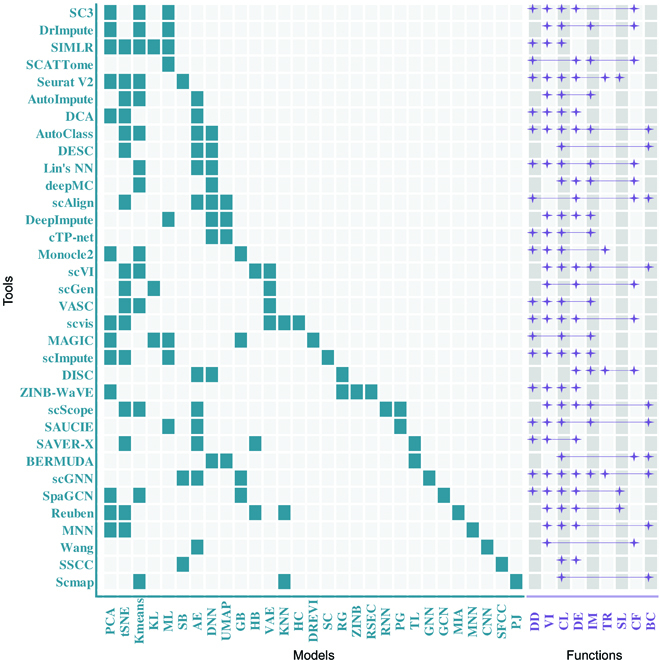
ML and DL techniques used in single-cell sequencing data analysis. Analysis tools are represented on the vertical axis, models are shown on the horizontal axis in green, and functions are implemented by tools in purple. KL, kernel learning; ML, metric learning; SB, statistic-based methods; AE, autoencoder; DNN, deep neural network; GB, graph-based methods; HB, hierarchical Bayesian; VAE, variational autoencoder; KNN, *k*-nearest neighbor; HC, hierarchical clustering; DREVI, density reweighted visualization; SC, spectral clustering; RG, regression; ZINB, zero-inflated negative binomial; RSEC, resampling-based sequential ensemble clustering; RNN, recurrent neural network; PG, PhenoGraph, a graph-based method; TL, transfer learning; GNN, graph neural network; GCN, graph convolutional network; MIA, multimodal intersection analysis; MNN, mutual nearest neighbors; CNN, convolutional neural network; SFCC, subsampling–featuring–clustering–classification; PJ, projection algorithm; DD, data denoising; VI, visualization; CL, clustering; DE, differential expression genes analysis; IM, imputation; TR, trajectory analysis; SL, spatial location; CF, classification; BC, batch correction.

Both ML and DL technologies have widely been used in single-cell sequencing data analysis. It is vital for guiding the development of feature extraction on cancer single cells and drug compounds. To compare the technical application gap between single-cell sequencing data analysis and cancer single-cell drug sensitivity analysis, we systematically summarized the single-cell sequencing data analysis methods (Fig. 6).

First, due to the sparsity of single-cell data, most algorithms will perform data reduction and denoising steps before inputting data. The most used dimensionality reduction method is principal components analysis (PCA) [70], the most used denoising method is Seurat, and some other studies do not carry out dimensionality reduction and denoising steps but directly put the original data into the AE neural network for feature extraction. In the field of single-cell genomics, autoencoders have been used for imputation, dimensionality reduction, and representation learning. On the aspect of visualization, t-distributed stochastic neighbor embedding (t-SNE) [71] leads the way. We notice that almost all algorithms use t-SNE, and fewer tools use uniform manifold approximation and projection for dimension reduction (UMAP) [72] or PCA to achieve similar results. In terms of imputation, most of the tools use AE or VAE and their variants. Lotfollahi et al. [20] provided a computing method called scGen that combined VAE and latent space vector arithmetics to capture features, which can distinguish responding from nonresponding genes and cells. The main functions of AE are feature extraction and denoising. Autoencoders force the network to extract useful features of data, as the bottleneck layer makes it learn the perfect reconstruction. Besides, reconstructing the data is often interpreted as denoising because the unimportant variations are automatically left out. Some methods are based on zero-inflated negative binomial and DNN. For example, DISC, a DL network together with semisupervised learning, is used to infer gene structure and expression obscured by dropouts and improve cell type identification for sparse scRNA-seq data [73].

Among all tasks, cluster analysis is the most extensive in single-cell sequencing data. Most of the tool algorithms have performed clustering analysis on single-cell sequencing data, and the most commonly used clustering method is *k*-means. Moreover, there are also some classical ML methods used for cluster analysis, such as spectral clustering or statistic-based method. In addition to cluster analysis, there are also differential expression gene analysis, trajectory analysis, spatial location, and batch correction in single-cell analysis tasks. Table 3 categorizes the ML and DL methods by function.

**Table 3.** ML and DL methods for single-cell sequencing data classified by function.

Functions	Methods
Data denoising	SC3 [99],SIMLR [98], SCATTome [96], Seurat V2 [100], DCA [112], AutoClass [126], Lin's NN [6], scAlign [117], cTP-net [120], Monocle2 [97], VASC [108], scvis [109], MAGIC [104], scImpute [110], ZINB-WaVE [105], SAUCIE [115], SAVER-X [113], scGNN [125], SpaGCN [124]
Visualization	SC3 [99], DrImpute [101], SIMLR [98], Seurat V2 [100], AutoImpute [102], DCA [112], AutoClass [126], Lin's NN [6], DeepImpute [119], cTP-net [120], Monocle2 [97], scVI [103], scGen [20], VASC [108], scvis [109], scImpute [110], ZINB-WaVE [105], scScope [111], SAUCIE [115], SAVER-X [113], scGNN [125], SpaGCN [124], Reuben [122], MNN [107], Wang [114]
Clustering	SC3 [99], DrImpute [101], SIMLR [98], Seurat V2 [100], AutoImpute [102], DCA [112], AutoClass [126], DESC [121], Lin's NN [6], deepMC [123], DeepImpute [119], cTP-net [120], Monocle2 [97], scVI [103], VASC [108], scvis [109], MAGIC [104], scImpute [110], ZINB-WaVE [105], scScope [111], SAUCIE [115], BERMUDA [118], scGNN [125], SpaGCN[124], Reuben [122], MNN [106], SSCC [116], Scmap [107]
Differential expression gene analysis	SC3 [99], SCATTome [96], Seurat V2 [100], DCA [112], AutoClass [126], deepMC [123], scAlign [117], DeepImpute [119], scVI [103], scGen [19], scvis [109], scImpute [110], DISC [73], ZINB-WaVE [105], scScope [111], SAVER-X [113], scGNN [125], SpaGCN [124], Reuben [122], MNN [106], SSCC [116]
Imputation	DrImpute [101], SCATTome [96], AutoImpute [102], AutoClass [126], Lin's NN [6], deepMC [123], DeepImpute [119], cTP-net [120], scVI [103], VASC [108], MAGIC [104], scImpute [110], scVI [103], VASC [108], MAGIC [104], scImpute [110], DISC [73], scScope [111], SAUCIE [115], scGNN [125]
Trajectory analysis	Seurat V2 [100], Monocle2 [97], DISC [73], scGNN [125]
Spatial location	Seurat V2 [100], SpaGCN [124], Reuben [122]
Classification	SC3 [99], DrImpute [101], SCATTome [96], Lin's NN [6], deepMC [123], scAlign [117], scGen [20], scvis [109], DISC [73], BERMUDA [118], Wang [114]
Batch correction	AutoClass [126], DESC [121], scAlign [117], scVI [103], scScope [111], SAUCIE [115], BERMUDA [118], scGNN [125],MNN [106], Scmap [107]

We found that in both sequence data analysis and image data analysis, algorithms are converging from ML to DL, and the focus of research has gradually shifted from single-cell clustering analysis to a more extensive exploration of the significance of biological internal mechanisms. We show the details of each method in Table 4, hoping that this summary will provide some valuable reference for beginners and also inspire innovative use of ML methods to address a broader range of new challenges in precision medicine.

**Table 4.** Detailed information on the ML and DL methods applied in single-cell sequencing data analysis.

Method	Year	Platform	Code availability	DOI
SCATTome [96]	2016	R	https://github.com/bvnlab/SCATTome	10.1038/leu.2015.361
Monocle2 [97]	2017	R	https://github.com/cole-trapnell-lab/monocle-release	10.1038/nmeth.4402
SIMLR [98]	2017	Python	https://github.com/bowang87/SIMLR_PY	10.1038/nmeth.4207
SC3 [99]	2017	R	http://bioconductor.org/packages/SC3/	10.1038/nmeth.4236
Seurat V2 [100]	2017	R	https://satijalab.org/seurat/	10.1038/nbt.4096
Lin's NN [6]	2017	Python	https://github.com/fchollet/keras	10.1093/nar/gkx681
DrImpute [101]	2018	R	https://github.com/gongx030/DrImpute	10.1186/s12859-018-2226-y
AutoImpute [102]	2018	Python, R	https://github.com/divyanshu-talwar/AutoImpute	10.1038/s41598-018-34688-x
scVI [103]	2018	Python	https://github.com/YosefLab/scVI	10.1038/s41592-018-0229-2
MAGIC [104]	2018	Python, R, Matlab	https://github.com/KrishnaswamyLab/MAGIC	10.1016/j.cell.2018.05.061
ZINB-WaVE [105]	2018	R	https://github.com/drisso/zinbwave	10.1038/s41467-017-02554-5
MNN [106]	2018	R	https://github.com/MarioniLab/MNN2017	doi.org/10.1038/nbt.4091
Scmap [107]	2018	R	http://bioconductor.org/packages/scmap	10.1038/nmeth.4644
VASC [108]	2018	Python	https://github.com/wang-research/VASC	10.1016/j.gpb.2018.08.003
scvis [109]	2018	Python	https://bitbucket.org/jerry00/scvis-dev	10.1038/s41467-018-04368-5
scImpute [110]	2018	R	https://github.com/Vivianstats/scImpute	10.1038/s41467-018-03405-7
scScope [111]	2019	Python	https://github.com/AltschulerWu-Lab/scScope	10.1038/s41592-019-0353-7
DCA [112]	2019	Python	https://github.com/theislab/dca	10.1038/s41467-018-07931-2
SAVER-X [113]	2019	R	https://github.com/jingshuw/SAVERX	10.1038/s41592-019-0537-1
Wang [114]	2019	Python	https://github.com/opnumten	10.1016/j.compbiomed.2019.04.006
SAUCIE [115]	2019	Python	https://github.com/KrishnaswamyLab/SAUCIE	10.1038/s41592-019-0576-7
SSCC [116]	2019	R	https://github.com/Japrin/sscClust	10.1016/j.gpb.2018.10.003
scAlign [117]	2019	R	https://github.com/quon-titative-biology	10.1186/s13059-019-1766-4
BERMUDA [118]	2019	Python	https://github.com/txWang/BERMUDA	10.1186/s13059-019-1764-6
scGen [20]	2019	Python	https://github.com/theislab/scgen	10.1038/s41592-019-0494-8
DeepImpute [119]	2019	Python	https://github.com/lanagarmire/DeepImpute	10.1186/s13059-019-1837-6
cTP-net [120]	2020	Python, R	https://github.com/zhouzilu/cTPnet	10.1038/s41467-020-14391-0
DISC [73]	2020	R	https://github.com/xie-lab/DISC	10.1186/s13059-020-02083-3
DESC [121]	2020	Python	https://eleozzr.github.io/desc/	10.1038/s41467-020-15851-3
Reuben [122]	2020	NA	NA	10.1038/s41587-019-0392-8
deepMC [123]	2020	NA	NA	10.1089/cmb.2019.0278
SpaGCN [124]	2021	Python	https://github.com/jianhuupenn/SpaGCN	10.1038/s41592-021-01255-8
scGNN [125]	2021	Python, R	https://github.com/juexinwang/scGNN	10.1038/s41467-021-22197-x
AutoClass [126]	2022	Python	https://github.com/datapplab/AutoClass	10.1038/s41467-022-29576-y

### Suggestions for the development of drug research methods at the single-cell level

According to the single-cell data analysis methods in Table 4, we can see the power of DL in various aspects, such as predicting protein results and extracting features. Due to the small amount of single-cell data containing cancer labels, the power of DL methods is still underexplored in single-cell drug sensitivity prediction, so how to use cancer single-cell data to predict drug sensitivity has become an urgent task in current cancer data analysis. Here, we give 3 suggestions for future method development at the single-cell drug level: (a) Drug prediction algorithms at the single-cell level can further explore the application potential of DL and make full use of its algorithmic advantages to promote the progress of cancer single-cell research. (b) The development of DL methods should pay attention to model interpretability. Although traditional DL models can predict drug responses for cell lines, these models are less transparent and interpretable, and the underlying mechanisms of drug resistance are often obscured by the black box of DL model predictions. Enhancing the interpretability of models is therefore essential to leverage DL approaches. (c) We suggest that drug study based on single cell should make full use of cancer cell line data information. Although there is a lack of a large amount of cancer single-cell sequencing data with drug response labels, which is essential for the training of DL models, a large amount of cancer cell line data exists. Future research approaches can make full use of the information from bulk RNA-seq data, enabling this information to aid in the training of cancer single-cell data. The powerful feature extraction capabilities of DL can help research on drug repurposing. If the sensitivity of single cells to drugs can be successfully predicted, it will be expected to develop combination drug delivery mechanisms for individual cancer cells and achieve precision medicine. In conclusion, DL methods for predicting drug sensitivity have a lot of urgent research topics in single-cell data analysis.

## Conclusion and Future Directions

Tumors are highly heterogeneous, which accounts for the different sensitivity of the subpopulations. In the previous study based on bulk data to predict the sensitivity at the tissue level, lots of expressions are masked or covered by the dominant subpopulations, which results in the loss of heterogeneity information. Tumor heterogeneity is considered the main reason for drug resistance. So the development of single-cell technology provides us the ability to profile cancer heterogeneity at single-cell resolution and offer us their expression patterns. Predicting the response of specific drugs in different cancer subpopulations will allow for personalized medicine and guide the drug combination design.

Sing-cell sequencing data can reveal cell-specific gene expression patterns from intratumoral heterogeneity, and DL methods could take advantage of the capabilities to analyze large-scale data and extract information on gene–drug correlation at the single-cell level. Although there are not enough single-cell sequencing data and cells’ responses after drug exposure, and lack of methods that can drug respond from single-cell gene expression sequences alone with high applicability and accuracy. Studies could integrate single-cell multiomic data and use multiple data resources with gene expression and drug response to predict the drug sensitivities for cancer subpopulations according to their scRNA-seq data. Also, it would be worth considering incorporating DNA into the model, which will provide some substantial novelty as well. Relative studies and results may provide a possible cost-efficient way when clinicians will make decisions on drug treatments for various groups of cancer patients with different cell-specific gene expression profiles.

The combination of scRNA-seq and DL technology will assist the practical solutions for clinical trials in the future, in addition to shedding more light on uncovering the molecular mechanisms underlying. It has the potential to greatly affect everyone who is affected by cancer, and it is looking at a very relevant problem and has the ability to lower cancer spending on the study and the cancer patients themselves. With the releasing of more cancer cell sample data in the future, researchers would use known information to figure out which types of cancer cells are drug resistant or sensitive, so as to transform current cancer care into more effective care that has the potential to help current and future cancer patients. The combination of single-cell data and DL techniques has successfully inferred a high diversity of tumor and immune cell populations and has greatly accelerated the discovery of new pathogenesis and cancer therapies. Another growing trend is the migration of DL models from predictable and interpretable to more actionable directions, such as drug repurposing and drug combination therapy.

## Data Availability

All data collected in the paper can be accessed by the GEO numbers in Table 1.

## References

[1] Ben-David U, Siranosian B, Ha G, Tang H, Oren Y, Hinohara K, Strathdee CA, Dempster J, Lyons NJ, Burns R, et al. Genetic and transcriptional evolution alters cancer cell line drug response. Nature. 2018;560(7718):325–330. 30089904 10.1038/s41586-018-0409-3 PMC6522222

[2] Patel L, Shukla T, Huang X, Ussery DW, Wang S. Machine learning methods in drug discovery. Molecules. 2020;25(22):5277. 33198233 10.3390/molecules25225277 PMC7696134

[3] Scheeder C, Heigwer F, Boutros M. Machine learning and image-based profiling in drug discovery. Curr Opin Syst Biol. 2018;10:43–52. 30159406 10.1016/j.coisb.2018.05.004 PMC6109111

[4] Yan R, Fan C, Yin Z, Wang T, Chen X. Potential applications of deep learning in single-cell RNA sequencing analysis for cell therapy and regenerative medicine. Stem Cells. 2021;39(5):511–521. 33587792 10.1002/stem.3336

[5] Zheng J, Wang K. Emerging deep learning methods for single-cell RNA-seq data analysis. Quant Biol. 2019;7(4):247–254.

[6] Lin C, Jain S, Kim H, Bar-Joseph Z. Using neural networks for reducing the dimensions of single-cell RNA-Seq data. Nucleic Acids Res. 2017;45(17):e156. 28973464 10.1093/nar/gkx681 PMC5737331

[7] Yang W, Soares J, Greninger P, Edelman EJ, Lightfoot H, Forbes S, Bindal N, Beare D, Smith JA, Thompson IR, et al. Genomics of drug sensitivity in cancer (GDSC): A resource for therapeutic biomarker discovery in cancer cells. Nucleic Acids Res. 2012;41(D1):D955–D961. 23180760 10.1093/nar/gks1111 PMC3531057

[8] Barretina J, Caponigro G, Stransky N, Venkatesan K, Margolin AA, Kim S, Wilson CJ, Lehár J, Kryukov GV, Sonkin D, et al. The cancer cell line encyclopedia enables predictive modelling of anticancer drug sensitivity. Nature. 2012;483(7391):603–607. 22460905 10.1038/nature11003 PMC3320027

[9] Rees MG, Seashore-Ludlow B, Cheah JH, Adams DJ, Price EV, Gill S, Javaid S, Coletti ME, Jones VL, Bodycombe NE, et al. Correlating chemical sensitivity and basal gene expression reveals mechanism of action. Nat Chem Biol. 2016;12(2):109–116. 26656090 10.1038/nchembio.1986 PMC4718762

[10] Subramanian A, Narayan R, Corsello SM, Peck DD, Natoli TE, Lu X, Gould J, Davis JF, Tubelli AA, Asiedu JK, et al. A next generation connectivity map: L1000 platform and the first 1,000,000 profiles. Cell. 2017;171(6):1437–1452.e17. 29195078 10.1016/j.cell.2017.10.049 PMC5990023

[11] Adam G, Rampášek L, Safikhani Z, Smirnov P, Haibe-Kains B, Goldenberg A. Machine learning approaches to drug response prediction: Challenges and recent progress. NPJ Precis Oncol. 2020;4(1):19. 32566759 10.1038/s41698-020-0122-1 PMC7296033

[12] Vamathevan J, Clark D, Czodrowski P, Dunham I, Ferran E, Lee G, Li B, Madabhushi A, Shah P, Spitzer M, et al. Applications of machine learning in drug discovery and development. Nat Rev Drug Discov. 2019;18(6):463–477. 30976107 10.1038/s41573-019-0024-5 PMC6552674

[13] Biau G, Scornet E. A random forest guided tour. TEST. 2016;25(2):197–227.

[14] Tibshirani R. Regression shrinkage and selection via the lasso. J R Stat Soc Ser B Methodol. 1996;58(1):267–288.

[15] Schuldt, C. Laptev I, Caputo B. Recognizing human actions: A local SVM approach. Paper presented at: Proceedings of the 17th International Conference on Pattern Recognition; 2004; Cambridge, UK. p. 32–36.

[16] Wong CH, Siah KW, Lo AW. Estimation of clinical trial success rates and related parameters. Biostatistics. 2019;20(2):273–286. 29394327 10.1093/biostatistics/kxx069 PMC6409418

[17] Wu Z, Lawrence PJ, Ma A, Zhu J, Xu D, Ma Q. Single-cell techniques and deep learning in predicting drug response. Trends Pharmacol Sci. 2020;41(12):1050–1065. 33153777 10.1016/j.tips.2020.10.004 PMC7669610

[18] Flores M, Liu Z, Zhang T, Hasib MM, Chiu YC, Ye Z, Paniagua K, Jo S, Zhang J, Gao SJ, et al. Deep learning tackles single-cell analysis—A survey of deep learning for scRNA-seq analysis. Brief Bioinform. 2022;23(1):Article bbab531. 34929734 10.1093/bib/bbab531 PMC8769926

[19] Filipp FV. Opportunities for artificial intelligence in advancing precision medicine. Curr Genet Med Rep. 2019;7(4):208–213. 31871830 10.1007/s40142-019-00177-4 PMC6927552

[20] Lotfollahi M, Wolf FA, Theis FJ. scGen predicts single-cell perturbation responses. Nat Methods. 2019;16(8):715–721. 31363220 10.1038/s41592-019-0494-8

[21] Wang C, June Choi H, Woodbury LS, Lee K. Deep learning-based subcellular phenotyping of protrusion dynamics reveals fine differential drug responses at subcellular and single-cell levels. Biophys J. 2022;121(3):529a.

[22] Sun J, Tárnok A, Su X. Deep learning-based single-cell optical image studies. Cytometry A. 2020;97(3):226–240. 31981309 10.1002/cyto.a.23973

[23] Sonnenburg S, Rätsch G, Schäfer C, Schölkopf B. Large scale multiple kernel learning. J Mach Learn Res. 2006;7:1531–1565.

[24] Mitchell TM, Bayesian learning. *Machine learning [M]*. New York: McGraw-Hill; 1997. p. 154–200.

[25] Quesada I, Grossmann IE. An LP/NLP based branch and bound algorithm for convex MINLP optimization problems. Comput Chem Eng. 1992;16(10–11):937–947.

[26] Sagi O, Rokach L. Ensemble learning: A survey. Wiley Interdiscip Rev Data Min Knowl Discov. 2018;8(4):Article e1249.

[27] Dietterich TG. Ensemble learning. The handbook of brain theory and neural networks. Arbib MA. 2002;2(1):110–125.

[28] Bao S, Li K, Yan C, Zhang Z, Qu J, Zhou M. Deep learning-based advances and applications for single-cell RNA-sequencing data analysis. Brief Bioinform. 2022;23(1):Article bbab473. 34849562 10.1093/bib/bbab473

[29] Siu DM, Lee KCM, Lo MCK, Stassen SV, Wang M, Zhang IZQ, So HKH, Chan GCF, Cheah KSE, Wong KKY, et al. Deep-learning-assisted biophysical imaging cytometry at massive throughput delineates cell population heterogeneity. Lab Chip. 2020;20(20):3696–3708. 32935707 10.1039/d0lc00542h

[30] Chen H, Engkvist O, Wang Y, Olivecrona M, Blaschke T. The rise of deep learning in drug discovery. Drug Discov Today. 2018;23(6):1241–1250. 29366762 10.1016/j.drudis.2018.01.039

[31] Li G, Sastry Hari SK, Sullivan M, Tsai T, Pattabiraman K, Emer J, Keckler SW. Understanding error propagation in deep learning neural network (DNN) accelerators and applications. Paper presented at: Networking, Storage and Analysis: Proceedings of the International Conference for High Performance Computing; 2017 Nov 12–17; Denver, Colorado p. 1–12.

[32] Gupta R, Srivastava D, Sahu M, Tiwari S, Ambasta RK, Kumar P. Artificial intelligence to deep learning: Machine intelligence approach for drug discovery. Mol Divers. 2021;25(3):1315–1360. 33844136 10.1007/s11030-021-10217-3 PMC8040371

[33] Lavecchia A. Deep learning in drug discovery: Opportunities, challenges and future prospects. Drug Discov Today. 2019;24(10):2017–2032. 31377227 10.1016/j.drudis.2019.07.006

[34] Leven O. The renaissance of phenotypic research: Serendipitous pharmaceutical discovery is making a comeback through HCS. Genet Eng Biotechnol News. 2014;34(7):26–27.

[35] Koumakis L. Deep learning models in genomics; are we there yet? Comput Struct Biotechnol J. 2020;18:1466–1473. 32637044 10.1016/j.csbj.2020.06.017 PMC7327302

[36] Sun Y, Sheng Z, Ma C, Tang K, Zhu R, Wu Z, Shen R, Feng J, Wu D, Huang D, et al. Combining genomic and network characteristics for extended capability in predicting synergistic drugs for cancer. Nat Commun. 2015;6(1):Article 8481. 26412466 10.1038/ncomms9481 PMC4598846

[37] Pan C, Schoppe O, Parra-Damas A, Cai R, Todorov MI, Gondi G, von Neubeck B, Böğürcü-Seidel N, Seidel S, Sleiman K, et al. Deep learning reveals cancer metastasis and therapeutic antibody targeting in the entire body. Cell. 2019;179(7):1661–1676.e19. 31835038 10.1016/j.cell.2019.11.013 PMC7591821

[38] Wang Y, Yang Y, Chen S, Wang J. DeepDRK: A deep learning framework for drug repurposing through kernel-based multi-omics integration. Brief Bioinform. 2021;22(5):Article bbab048. 33822890 10.1093/bib/bbab048

[39] He X, Folkman L, Borgwardt K. Kernelized rank learning for personalized drug recommendation. Bioinformatics. 2018;34(16):2808–2816. 29528376 10.1093/bioinformatics/bty132 PMC6084606

[40] Zeng X, Zhu S, Liu X, Zhou Y, Nussinov R, Cheng F. deepDR: A network-based deep learning approach to in silico drug repositioning. Bioinformatics. 2019;35(24):5191–5198. 31116390 10.1093/bioinformatics/btz418 PMC6954645

[41] Yu T-H, Su BH, Battalora LC, Liu S, Tseng YJ. Ensemble modeling with machine learning and deep learning to provide interpretable generalized rules for classifying CNS drugs with high prediction power. Brief Bioinform. 2022;23(1):Article bbab377. 34530437 10.1093/bib/bbab377 PMC8769704

[42] Altae-Tran H, Ramsundar B, Pappu AS, Pande V. Low data drug discovery with one-shot learning. ACS Cent Sci. 2017;3(4):283–293. 28470045 10.1021/acscentsci.6b00367 PMC5408335

[43] Issa NT, Stathias V, Schürer S, Dakshanamurthy S. Machine and deep learning approaches for cancer drug repurposing. Semin Cancer Biol. 2021;68:132–142. 31904426 10.1016/j.semcancer.2019.12.011 PMC7723306

[44] Costello JC, Heiser LM, Georgii E, Gönen M, Menden MP, Wang NJ, Bansal M, Ammad-ud-din M, Hintsanen P, Khan SA, et al. A community effort to assess and improve drug sensitivity prediction algorithms. Nat Biotechnol. 2014;32(12):1202–1212. 24880487 10.1038/nbt.2877 PMC4547623

[45] Zhang F, Wang M, Xi J, Yang J, Li A. A novel heterogeneous network-based method for drug response prediction in cancer cell lines. Sci Rep. 2018;8(1):Article 3355. 29463808 10.1038/s41598-018-21622-4 PMC5820329

[46] Sharifi-Noghabi H, Zolotareva O, Collins CC, Ester M. MOLI: Multi-omics late integration with deep neural networks for drug response prediction. Bioinformatics. 2019;35(14):i501–i509. 31510700 10.1093/bioinformatics/btz318 PMC6612815

[47] Ma J, Yu MK, Fong S, Ono K, Sage E, Demchak B, Sharan R, Ideker T. Using deep learning to model the hierarchical structure and function of a cell. Nat Methods. 2018;15(4):290–298. 29505029 10.1038/nmeth.4627 PMC5882547

[48] Choi J, Park S, Ahn J. RefDNN: A reference drug based neural network for more accurate prediction of anticancer drug resistance. Sci Rep. 2020;10(1):Article 1861. 32024872 10.1038/s41598-020-58821-x PMC7002431

[49] Liu P, Li H, Li S, Leung KS. Improving prediction of phenotypic drug response on cancer cell lines using deep convolutional network. BMC Bioinformatics. 2019;20(1):Article 408. 31357929 10.1186/s12859-019-2910-6 PMC6664725

[50] Nguyen T, Nguyen GTT, Nguyen T, Le DH. Graph convolutional networks for drug response prediction. IEEE/ACM Trans Comput Biol Bioinform. 2021;19(1):146–154. 10.1109/TCBB.2021.3060430 33606633

[51] Liu Q, Hu Z, Jiang R, Zhou M. DeepCDR: A hybrid graph convolutional network for predicting cancer drug response. Bioinformatics. 2020;36(Suppl_2):i911–i918. 33381841 10.1093/bioinformatics/btaa822

[52] Rampášek L, Hidru D, Smirnov P, Haibe-Kains B, Goldenberg A. Dr. VAE: Improving drug response prediction via modeling of drug perturbation effects. Bioinformatics. 2019;35(19):3743–3751. 30850846 10.1093/bioinformatics/btz158 PMC6761940

[53] Wen M, Zhang Z, Niu S, Sha H, Yang R, Yun Y, Lu H. Deep-learning-based drug–target interaction prediction. J Proteome Res. 2017;16(4):1401–1409. 28264154 10.1021/acs.jproteome.6b00618

[54] Wan F, Zhu Y, Hu H, Dai A, Cai X, Chen L, Gong H, Xia T, Yang D, Wang MW, et al. DeepCPI: A deep learning-based framework for large-scale in silico drug screening. Genom Proteom Bioinform. 2019;17(5):478–495. 10.1016/j.gpb.2019.04.003 PMC7056933 32035227

[55] Huang K, Fu T, Glass LM, Zitnik M, Xiao C, Sun J. DeepPurpose: A deep learning library for drug–target interaction prediction. Bioinformatics. 2020;36(22–23):5545–5547. 10.1093/bioinformatics/btaa1005 PMC8016467 33275143

[56] Lee I, Keum J, Nam H. DeepConv-DTI: Prediction of drug-target interactions via deep learning with convolution on protein sequences. PLOS Comput Biol. 2019;15(6):Article e1007129. 31199797 10.1371/journal.pcbi.1007129 PMC6594651

[57] Rifaioglu AS, Nalbat E, Atalay V, Martin MJ, Cetin-Atalay R, Doğan T. DEEPScreen: High performance drug–target interaction prediction with convolutional neural networks using 2-D structural compound representations. Chem Sci. 2020;11(9):2531–2557. 33209251 10.1039/c9sc03414e PMC7643205

[58] Yuan Y, Bar-Joseph Z. Deep learning for inferring gene relationships from single-cell expression data. Proc Natl Acad Sci USA. 2019;116(52):27151–27158. 31822622 10.1073/pnas.1911536116 PMC6936704

[59] Karimi M, Wu D, Wang Z, Shen Y. DeepAffinity: Interpretable deep learning of compound–protein affinity through unified recurrent and convolutional neural networks. Bioinformatics. 2019;35(18):3329–3338. 30768156 10.1093/bioinformatics/btz111 PMC6748780

[60] Yan J, Bhadra P, Li A, Sethiya P, Qin L, Tai HK, Wong KH, Siu SWI. Deep-AmPEP30: Improve short antimicrobial peptides prediction with deep learning. Mol Ther Nucleic Acids. 2020;20:882–894. 32464552 10.1016/j.omtn.2020.05.006 PMC7256447

[61] Preuer K, Lewis RPI, Hochreiter S, Bender A, Bulusu KC, Klambauer G. DeepSynergy: Predicting anti-cancer drug synergy with deep learning. Bioinformatics. 2018;34(9):1538–1546. 29253077 10.1093/bioinformatics/btx806 PMC5925774

[62] Zeng X, Zhu S, Lu W, Liu Z, Huang J, Zhou Y, Fang J, Huang Y, Guo H, Li L, et al. Target identification among known drugs by deep learning from heterogeneous networks. Chem Sci. 2020;11(7):1775–1797. 34123272 10.1039/c9sc04336e PMC8150105

[63] Zitnik M, Agrawal M, Leskovec J. Modeling polypharmacy side effects with graph convolutional networks. Bioinformatics. 2018;34(13):i457–i466. 29949996 10.1093/bioinformatics/bty294 PMC6022705

[64] Deng Y, Xu X, Qiu Y, Xia J, Zhang W, Liu S. A multimodal deep learning framework for predicting drug–drug interaction events. Bioinformatics. 2020;36(15):4316–4322. 32407508 10.1093/bioinformatics/btaa501

[65] Kandaswamy C, Silva LM, Alexandre LA, Santos JM. High-content analysis of breast cancer using single-cell deep transfer learning. J Biomol Screen. 2016;21(3):252–259. 26746583 10.1177/1087057115623451

[66] Pavillon N, Hobro AJ, Akira S, Smith NI. Noninvasive detection of macrophage activation with single-cell resolution through machine learning. Proc Natl Acad Sci USA. 2018;115(12):E2676–E2685. 29511099 10.1073/pnas.1711872115 PMC5866539

[67] Kobayashi H, Lei C, Wu Y, Mao A, Jiang Y, Guo B, Ozeki Y, Goda K. Label-free detection of cellular drug responses by high-throughput bright-field imaging and machine learning. Sci Rep. 2017;7(1):Article 12454. 28963483 10.1038/s41598-017-12378-4 PMC5622112

[68] Yanagisawa K, Toratani M, Asai A, Konno M, Niioka H, Mizushima T, Satoh T, Miyake J, Ogawa K, Vecchione A, et al. Convolutional neural network can recognize drug resistance of single cancer cells. Int J Mol Sci. 2020;21(9):Article 3166. 32365822 10.3390/ijms21093166 PMC7246790

[69] Mencattini A, di Giuseppe D, Comes MC, Casti P, Corsi F, Bertani FR, Ghibelli L, Businaro L, di Natale C, Parrini MC, et al. Discovering the hidden messages within cell trajectories using a deep learning approach for in vitro evaluation of cancer drug treatments. Sci Rep. 2020;10(1):Article 7653. 32376840 10.1038/s41598-020-64246-3 PMC7203117

[70] Yang J, Zhang D, Frangi AF, Yang J-y. Two-dimensional PCA: A new approach to appearance-based face representation and recognition. IEEE Trans Pattern Anal Mach Intell. 2004;26(1):131–137. 15382693 10.1109/tpami.2004.1261097

[71] Van der Maaten L, Hinton G. Visualizing data using t-SNE. J Mach Learn Res. 2008;9(86):2579–2605.

[72] McInnes, L, Healy J, Melville J. Umap: Uniform manifold approximation and projection for dimension reduction. arXiv. 2018. https://arxiv.org/abs/1802.03426.

[73] He Y, Yuan H, Wu C, Xie Z. DISC: A highly scalable and accurate inference of gene expression and structure for single-cell transcriptomes using semi-supervised deep learning. Genome Biol. 2020;21(1):Article 170. 32650816 10.1186/s13059-020-02083-3 PMC7353747

[74] Bell CC, Fennell KA, Chan YC, Rambow F, Yeung MM, Vassiliadis D, Lara L, Yeh P, Martelotto LG, Rogiers A, et al. Targeting enhancer switching overcomes non-genetic drug resistance in acute myeloid leukaemia. Nat Commun. 2019;10(1):Article 2723. 31222014 10.1038/s41467-019-10652-9 PMC6586637

[75] Kim C, Gao R, Sei E, Brandt R, Hartman J, Hatschek T, Crosetto N, Foukakis T, Navin NE. Chemoresistance evolution in triple-negative breast cancer delineated by single-cell sequencing. Cell. 2018;173(4):879–893.e13. 29681456 10.1016/j.cell.2018.03.041 PMC6132060

[76] Wang Q, Guldner IH, Golomb SM, Sun L, Harris JA, Lu X, Zhang S. Single-cell profiling guided combinatorial immunotherapy for fast-evolving CDK4/6 inhibitor-resistant HER2-positive breast cancer. Nat Commun. 2019;10(1):Article 3817. 31444334 10.1038/s41467-019-11729-1 PMC6707314

[77] Rendeiro AF, Krausgruber T, Fortelny N, Zhao F, Penz T, Farlik M, Schuster LC, Nemc A, Tasnády S, Réti M, et al. Chromatin mapping and single-cell immune profiling define the temporal dynamics of ibrutinib response in CLL. Nat Commun. 2020;11(1):Article 577. 31996669 10.1038/s41467-019-14081-6 PMC6989523

[78] Rambow F, Rogiers A, Marin-Bejar O, Aibar S, Femel J, Dewaele M, Karras P, Brown D, Chang YH, Debiec-Rychter M, et al. Toward minimal residual disease-directed therapy in melanoma. Cell. 2018;174(4):843–855.e19. 30017245 10.1016/j.cell.2018.06.025

[79] Sharma A, Cao EY, Kumar V, Zhang X, Leong HS, Wong AML, Ramakrishnan N, Hakimullah M, Teo HMV, Chong FT, et al. Longitudinal single-cell RNA sequencing of patient-derived primary cells reveals drug-induced infidelity in stem cell hierarchy. Nat Commun. 2018;9(1):Article 4931. 30467425 10.1038/s41467-018-07261-3 PMC6250721

[80] Zhu D, Zhao Z, Cui G, Chang S, Hu L, See YX, Lim MGL, Guo D, Chen X, Poudel B, et al. Single-cell transcriptome analysis reveals estrogen signaling coordinately augments one-carbon, polyamine, and purine synthesis in breast cancer. Cell Rep. 2018;25(8):2285–2298.e4. 30463022 10.1016/j.celrep.2018.10.093

[81] Hinohara K, Wu HJ, Vigneau S, McDonald TO, Igarashi KJ, Yamamoto KN, Madsen T, Fassl A, Egri SB, Papanastasiou M, et al. KDM5 histone demethylase activity links cellular transcriptomic heterogeneity to therapeutic resistance. Cancer Cell. 2018;34(6):939–953.e9. 30472020 10.1016/j.ccell.2018.10.014 PMC6310147

[82] Grosselin K, Durand A, Marsolier J, Poitou A, Marangoni E, Nemati F, Dahmani A, Lameiras S, Reyal F, Frenoy O, et al. High-throughput single-cell ChIP-seq identifies heterogeneity of chromatin states in breast cancer. Nat Genet. 2019;51(6):1060–1066. 31152164 10.1038/s41588-019-0424-9

[83] Hamza B, Ng SR, Prakadan SM, Delgado FF, Chin CR, King EM, Yang LF, Davidson SM, DeGouveia KL, Cermak N, et al. Optofluidic real-time cell sorter for longitudinal CTC studies in mouse models of cancer. Proc Natl Acad Sci USA. 2019;116(6):2232–2236. 30674677 10.1073/pnas.1814102116 PMC6369805

[84] Wu H, Chen S, Yu J, Li Y, Zhang XY, Yang L, Zhang H, Hou Q, Jiang M, Brunicardi FC, et al. Single-cell transcriptome analyses reveal molecular signals to intrinsic and acquired paclitaxel resistance in esophageal squamous cancer cells. Cancer Lett. 2018;420:156–167. 29410067 10.1016/j.canlet.2018.01.059

[85] Gryder BE, Wu L, Woldemichael GM, Pomella S, Quinn TR, Park PMC, Cleveland A, Stanton BZ, Song Y, Rota R, et al. Chemical genomics reveals histone deacetylases are required for core regulatory transcription. Nat Commun. 2019;10(1):Article 3004. 31285436 10.1038/s41467-019-11046-7 PMC6614369

[86] Krivtsov AV, Evans K, Gadrey JY, Eschle BK, Hatton C, Uckelmann HJ, Ross KN, Perner F, Olsen SN, Pritchard T, et al. A menin-MLL inhibitor induces specific chromatin changes and eradicates disease in models of MLL-rearranged leukemia. Cancer Cell. 2019;36(6):660–673.e11. 31821784 10.1016/j.ccell.2019.11.001 PMC7227117

[87] Kong SL, Li H, Tai JA, Courtois ET, Poh HM, Lau DP, Haw YX, Iyer NG, Tan DSW, Prabhakar S, et al. Concurrent single-cell RNA and targeted DNA sequencing on an automated platform for comeasurement of genomic and transcriptomic signatures. Clin Chem. 2019;65(2):272–281. 30523199 10.1373/clinchem.2018.295717

[88] Kim K-T, Lee HW, Lee HO, Kim SC, Seo YJ, Chung W, Eum HH, Nam DH, Kim J, Joo KM, et al. Single-cell mRNA sequencing identifies subclonal heterogeneity in anti-cancer drug responses of lung adenocarcinoma cells. Genome Biol. 2015;16(1):Article 127. 26084335 10.1186/s13059-015-0692-3 PMC4506401

[89] Ho Y-J, Anaparthy N, Molik D, Mathew G, Aicher T, Patel A, Hicks J, Hammell MG. Single-cell RNA-seq analysis identifies markers of resistance to targeted BRAF inhibitors in melanoma cell populations. Genome Res. 2018;28(9):1353–1363. 30061114 10.1101/gr.234062.117 PMC6120620

[90] Jerby-Arnon L, Shah P, Cuoco MS, Rodman C, Su MJ, Melms JC, Leeson R, Kanodia A, Mei S, Lin JR, et al. A cancer cell program promotes T cell exclusion and resistance to checkpoint blockade. Cell. 2018;175(4):984–997.e24. 30388455 10.1016/j.cell.2018.09.006 PMC6410377

[91] Shaffer SM, Dunagin MC, Torborg SR, Torre EA, Emert B, Krepler C, Beqiri M, Sproesser K, Brafford PA, Xiao M, et al. Rare cell variability and drug-induced reprogramming as a mode of cancer drug resistance. Nature. 2017;546(7658):431–435. 28607484 10.1038/nature22794 PMC5542814

[92] Tirosh I, Izar B, Prakadan SM, Wadsworth MH II, Treacy D, Trombetta JJ, Rotem A, Rodman C, Lian C, Murphy G, et al. Dissecting the multicellular ecosystem of metastatic melanoma by single-cell RNA-seq. Science. 2016;352(6282):189–196. 27124452 10.1126/science.aad0501 PMC4944528

[93] Ocasio JK, Babcock B, Malawsky D, Weir SJ, Loo L, Simon JM, Zylka MJ, Hwang D, Dismuke T, Sokolsky M, et al. scRNA-seq in medulloblastoma shows cellular heterogeneity and lineage expansion support resistance to SHH inhibitor therapy. Nat Commun. 2019;10(1):Article 5829. 31863004 10.1038/s41467-019-13657-6 PMC6925218

[94] Schnepp PM, Shelley G, Dai J, Wakim N, Jiang H, Mizokami A, Keller ET. Single cell transcriptomics analysis identifies nuclear protein 1 as a regulator of docetaxel resistance in prostate cancer Cells. Mol Cancer Res. 2020;18(9):1290–1301. 32513898 10.1158/1541-7786.MCR-20-0051 PMC7483674

[95] Aissa AF, Islam ABMMK, Ariss MM, Go CC, Rader AE, Conrardy RD, Gajda AM, Rubio-Perez C, Valyi-Nagy K, Pasquinelli M, et al. Single-cell transcriptional changes associated with drug tolerance and response to combination therapies in cancer. Nat Commun. 2021;12(1):Article 1628. 33712615 10.1038/s41467-021-21884-z PMC7955121

[96] Mitra AK, Mukherjee UK, Harding T, Jang JS, Stessman H, Li Y, Abyzov A, Jen J, Kumar S, Rajkumar V, et al. Single-cell analysis of targeted transcriptome predicts drug sensitivity of single cells within human myeloma tumors. Leukemia. 2016;30(5):1094–1102. 26710886 10.1038/leu.2015.361

[97] Tian H, Biehs B, Warming S, Leong KG, Rangell L, Klein OD, de Sauvage FJ. A reserve stem cell population in small intestine renders Lgr5-positive cells dispensable. Nature. 2011;478(7368):255–259. 21927002 10.1038/nature10408 PMC4251967

[98] Yang W, Yuste R. In vivo imaging of neural activity. Nat Methods. 2017;14(4):349–359. 28362436 10.1038/nmeth.4230 PMC5903578

[99] Kiselev VY, Kirschner K, Schaub MT, Andrews T, Yiu A, Chandra T, Natarajan KN, Reik W, Barahona M, Green AR, et al. SC3: Consensus clustering of single-cell RNA-seq data. Nat Methods. 2017;14(5):483–486. 28346451 10.1038/nmeth.4236 PMC5410170

[100] Newman AM, Liu CL, Green MR, Gentles AJ, Feng W, Xu Y, Hoang CD, Diehn M, Alizadeh AA. Robust enumeration of cell subsets from tissue expression profiles. Nat Methods. 2015;12(5):453–457. 25822800 10.1038/nmeth.3337 PMC4739640

[101] Gong W, Kwak IY, Pota P, Koyano-Nakagawa N, Garry DJ. DrImpute: Imputing dropout events in single cell RNA sequencing data. BMC Bioinformatics. 2018;19(1):Article 220. 29884114 10.1186/s12859-018-2226-y PMC5994079

[102] Talwar D, Mongia A, Sengupta D, Majumdar A. AutoImpute: Autoencoder based imputation of single-cell RNA-seq data. Sci Rep. 2018;8(1):Article 16329. 30397240 10.1038/s41598-018-34688-x PMC6218547

[103] Lopez R, Regier J, Cole MB, Jordan MI, Yosef N. Deep generative modeling for single-cell transcriptomics. Nat Methods. 2018;15(12):1053–1058. 30504886 10.1038/s41592-018-0229-2 PMC6289068

[104] Van Dijk D, Sharma R, Nainys J, Yim K, Kathail P, Carr AJ, Burdziak C, Moon KR, Chaffer CL, Pattabiraman D, et al. Recovering gene interactions from single-cell data using data diffusion. Cell. 2018;174(3):716–729.e27. 29961576 10.1016/j.cell.2018.05.061 PMC6771278

[105] Risso D, Perraudeau F, Gribkova S, Dudoit S, Vert JP. A general and flexible method for signal extraction from single-cell RNA-seq data. Nat Commun. 2018;9(1):Article 284. 29348443 10.1038/s41467-017-02554-5 PMC5773593

[106] Jammula S, Katz-Summercorn AC, Li X, Linossi C, Smyth E, Killcoyne S, Biasci D, Subash VV, Abbas S, Blasko A, et al. Identification of subtypes of Barrett’s esophagus and esophageal adenocarcinoma based on DNA methylation profiles and integration of transcriptome and genome data. Gastroenterology. 2020;158(6):1682–1697.e1. 32032585 10.1053/j.gastro.2020.01.044 PMC7305027

[107] Kiselev VY, Yiu A, Hemberg M. Scmap: Projection of single-cell RNA-seq data across data sets. Nat Methods. 2018;15(5):359–362. 29608555 10.1038/nmeth.4644

[108] Wang D, Gu J. VASC: Dimension reduction and visualization of single-cell RNA-seq data by deep variational autoencoder. Genom Proteom Bioinform. 2018;16(5):320–331. 10.1016/j.gpb.2018.08.003 PMC6364131 30576740

[109] Ding J, Condon A, Shah SP. Interpretable dimensionality reduction of single cell transcriptome data with deep generative models. Nat Commun. 2018;9(1):Article 2002. 29784946 10.1038/s41467-018-04368-5 PMC5962608

[110] Li H, Courtois ET, Sengupta D, Tan Y, Chen KH, Goh JJL, Kong SL, Chua C, Hon LK, Tan WS, et al. Reference component analysis of single-cell transcriptomes elucidates cellular heterogeneity in human colorectal tumors. Nat Genet. 2017;49(5):708–718. 28319088 10.1038/ng.3818

[111] Deng Y, Bao F, Dai Q, Wu LF, Altschuler SJ. Scalable analysis of cell-type composition from single-cell transcriptomics using deep recurrent learning. Nat Methods. 2019;16(4):311–314. 30886411 10.1038/s41592-019-0353-7 PMC6774994

[112] Eraslan G, Simon LM, Mircea M, Mueller NS, Theis FJ. Single-cell RNA-seq denoising using a deep count autoencoder. Nat Commun. 2019;10(1):Article 390. 30674886 10.1038/s41467-018-07931-2 PMC6344535

[113] Wang J, Agarwal D, Huang M, Hu G, Zhou Z, Ye C, Zhang NR. Data denoising with transfer learning in single-cell transcriptomics. Nat Methods. 2019;16(9):875–878. 31471617 10.1038/s41592-019-0537-1 PMC7781045

[114] Wang W, Taft DA, Chen YJ, Zhang J, Wallace CT, Xu M, Watkins SC, Xing J. Learn to segment single cells with deep distance estimator and deep cell detector. Comput Biol Med. 2019;108:133–141. 31005005 10.1016/j.compbiomed.2019.04.006 PMC6781873

[115] Amodio M, van Dijk D, Srinivasan K, Chen WS, Mohsen H, Moon KR, Campbell A, Zhao Y, Wang X, Venkataswamy M, et al. Exploring single-cell data with deep multitasking neural networks. Nat Methods. 2019;16(11):1139–1145. 31591579 10.1038/s41592-019-0576-7 PMC10164410

[116] Ren X, Zheng L, Zhang Z. SSCC: A novel computational framework for rapid and accurate clustering large-scale single cell RNA-seq data. Genom Proteom Bioinform. 2019;17(2):201–210. 10.1016/j.gpb.2018.10.003 PMC6624216 31202000

[117] Johansen N, Quon G. scAlign: A tool for alignment, integration, and rare cell identification from scRNA-seq data. Genome Biol. 2019;20(1):Article 166. 31412909 10.1186/s13059-019-1766-4 PMC6693154

[118] Wang T, Johnson TS, Shao W, Lu Z, Helm BR, Zhang J, Huang K. BERMUDA:A novel deep transfer learning method for single-cell RNA sequencing batch correction reveals hidden high-resolution cellular subtypes. Genome Biol. 2019;20(1):Article 165. 31405383 10.1186/s13059-019-1764-6 PMC6691531

[119] Arisdakessian C, Poirion O, Yunits B, Zhu X, Garmire LX. DeepImpute: An accurate, fast, and scalable deep neural network method to impute single-cell RNA-seq data. Genome Biol. 2019;20(1):Article 211. 31627739 10.1186/s13059-019-1837-6 PMC6798445

[120] Zhou Z, Ye C, Wang J, Zhang NR. Surface protein imputation from single cell transcriptomes by deep neural networks. Nat Commun. 2020;11(1):Article 651. 32005835 10.1038/s41467-020-14391-0 PMC6994606

[121] Li X, Wang K, Lyu Y, Pan H, Zhang J, Stambolian D, Susztak K, Reilly MP, Hu G, Li M. Deep learning enables accurate clustering with batch effect removal in single-cell RNA-seq analysis. Nat Commun. 2020;11(1):Article 2338. 32393754 10.1038/s41467-020-15851-3 PMC7214470

[122] Moncada R, Barkley D, Wagner F, Chiodin M, Devlin JC, Baron M, Hajdu CH, Simeone DM, Yanai I. Integrating microarray-based spatial transcriptomics and single-cell RNA-seq reveals tissue architecture in pancreatic ductal adenocarcinomas. Nat Biotechnol. 2020;38(3):333–342. 31932730 10.1038/s41587-019-0392-8

[123] Mongia A, Sengupta D, Majumdar A. deepMc: Deep matrix completion for imputation of single-cell RNA-seq data. J Comput Biol. 2020;27(7):1011–1019. 31657645 10.1089/cmb.2019.0278

[124] Hu J. Li X, Coleman K, Schroeder A, Irwin DJ, Lee EB, Shinohara RT, Li M. Integrating gene expression, spatial location and histology to identify spatial domains and spatially variable genes by graph convolutional network. bioRxiv. 2020. https://www.biorxiv.org/content/10.1101/2020.11.30.405118v1. 10.1038/s41592-021-01255-8 34711970

[125] Wang J, Ma A, Chang Y, Gong J, Jiang Y, Qi R, Wang C, Fu H, Ma Q, Xu D. scGNN is a novel graph neural network framework for single-cell RNA-Seq analyses. Nat Commun. 2021;12(1):Article 1882. 33767197 10.1038/s41467-021-22197-x PMC7994447

[126] Li H, Brouwer CR, Luo W. A universal deep neural network for in-depth cleaning of single-cell RNA-seq data. Nat Commun. 2022;13(1):Article 1901. 35393428 10.1038/s41467-022-29576-y PMC8990021

[127] Chen J, Wang X, Ma A, Wang QE, Liu B, Li L, Xu D, Ma Q. Deep transfer learning of cancer drug responses by integrating bulk and single-cell RNA-seq data. Nat Commun. 2022;13(1):Article 6494. 36310235 10.1038/s41467-022-34277-7 PMC9618578

[128] Ji Y, Lotfollahi M, Alexander Wolf F, Theis FJ. Machine learning for perturbational single-cell omics. Cell Syst. 2021;12(6):522–537. 34139164 10.1016/j.cels.2021.05.016

[129] Dara S, Dhamercherla S, Jadav SS, Babu CHM, Ahsan MJ. Machine learning in drug discovery: A review. Artif Intell Rev. 2022;55(3):1947–1999. 34393317 10.1007/s10462-021-10058-4 PMC8356896

[130] Pepe G, Carrino C, Parca L, Helmer-Citterich M. Dissecting the genome for drug response prediction. Methods Mol Biol. 2022;2449(7):187–196. 35507263 10.1007/978-1-0716-2095-3_7

[131] Wang Z, Li H, Carpenter C, Guan Y. Challenge-enabled machine learning to drug-response prediction. AAPS J. 2020;22(5):Article 106. 32778984 10.1208/s12248-020-00494-5 PMC10176199

